# Hiccups as a Rare Presentation of Thyrotoxicosis Triaged by an Epidural Steroid Injection

**DOI:** 10.7759/cureus.16438

**Published:** 2021-07-17

**Authors:** Omar Al-Radideh, Iyad Farouji, Theodore DaCosta, Hossam Abed, Nicholas Baranestky

**Affiliations:** 1 Medical Education, Saint Michael’s Medical Center, Newark, USA; 2 Endocrinology and Diabetes, Saint Michael’s Medical Center, Newark, USA

**Keywords:** thyrotoxicosis, hiccups, epidural steroid injection, hyperthyrodism, thyroid-stimulating hormone (tsh)

## Abstract

Thyrotoxicosis manifests when excess levels of thyroid hormone act on different tissues throughout the body. Excess hormone levels can be related to endogenous production or exogenous ingestion and can present differently in patients. It has been theorized that high levels of thyroxine can irritate the neuroanatomical hiccup center leading to persistent hiccups. Although extremely rare, physicians should be aware of this entity to allow for proper diagnosis and management. Here, we discuss a rare case of thyrotoxicosis after an epidural steroid injection presenting with intractable hiccups.

## Introduction

Hiccups are considered intractable once they persist for more than 48 hours and can cause the patient significant distress [1]. The primary management of hiccups begins with the identification of the cause followed by appropriate treatment. Although gastric distention and alcohol consumption are common causes of hiccups, other rare disorders including metabolic syndromes should be on the differential in a patient presenting with hiccups [2,3]. Here, we present a very unique and rare presentation of thyrotoxicosis presenting with intractable hiccups after an epidural steroid injection.

## Case presentation

A 50-year-old African American male with a history of smoking and work-related traumatic back pain managed with physical exercise and epidural steroid injections presented to the emergency department complaining of intractable hiccups following an epidural steroid injection to the back one week prior to the hospital encounter. The patient described the hiccups as progressively worsening and interfering with his daily activities. He had not experienced any significant improvement with over-the-counter antacids or chlorpromazine use. The hiccups were not associated with nausea, vomiting, abdominal pain, or abdominal distention. History was also negative for tremors, nervousness, agitation, diarrhea, and ingestion of medications known to cause hiccups.

**Figure 1:**
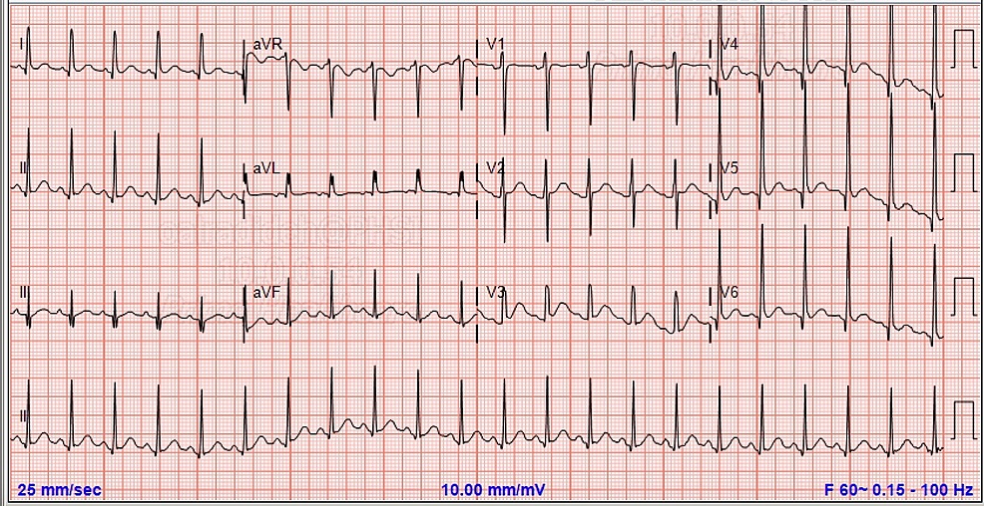
ECG showing sinus tachycardia. ECG: electrocardiogram

On examination, the patient was ill-appearing, in distress, oriented, and had a body mass index of 30.17 kg/m^2^. Vital signs revealed tachycardia (heart rate: 136 beats per minute), wide pulse pressure with a blood pressure of 157/61 mm Hg, respiratory rate of 15 breaths per minute, and a temperature of 98.1°F. He had diffused, soft but enlarged thyroid gland with an auscultatory bruit over the entire gland. His eye examination was negative for components of ophthalmopathy, such as bilateral proptosis. There were no physical signs of congestive heart failure or heart murmurs. The neurological examination was also unremarkable. Lab studies showed mild leukocytosis of 13.8 × 10^3^/uL and normal hemoglobin level and platelets count. The complete metabolic panel showed mild hypokalemia of 3.1 mmol/L (3.5-5.3 mmol/L), and isolated elevated alkaline phosphatase of 264 u/L (40-115 u/L). Other labs including calcium, magnesium, kidney function, liver function, troponin, and B-type natriuretic peptide were all within normal limits. Electrocardiography showed sinus tachycardia (Figure 1), with the echocardiogram showing no abnormality. Imaging studies including chest radiography and computed tomography scan of the head, chest, abdomen, and pelvis were unremarkable. Thyroid function testing revealed a very suppressed thyroid-stimulating hormone at less than 0.01 uIU/mL (0.4-4.5 uIU/mL), elevated free T4 >24 (0.8-1.8 ug/dL), and free T3 >32 (0.9-1.7 ng/mL). Thyroid ultrasound showed enlarged hypervascularity thyroid glands. In addition, the thyroid peroxidase antibodies were elevated at 37 IU/mL (0-34 IU/mL).

The patient was admitted to the medical floor and treated as a case of thyrotoxicosis without impending thyroid storm as the Burch-Wartofsky point scale was calculated to be 20 points. The patient was started on beta-blockers, atenolol 50 mg once a day, and methimazole 10 mg twice for one day and then switched to 15 mg once daily as per endocrine service recommendations along with replacement of potassium. The frequency and intensity of the hiccups improved during the hospital stay and the patient was discharged with a scheduled follow-up in the outpatient clinic to repeat thyroid function testing in two weeks.

## Discussion

Thyrotoxicosis is caused by an excess of thyroid hormone. The source of the hormone can be endogenous due to the oversecretion of T3 and T4 or due to the ingestion of synthetic thyroid hormone. Thyroid hormone is crucial and can affect all the organ systems in the body by directly affecting the basal metabolic rate and tissue thermogenesis through the upregulation of alpha-adrenergic receptors leading to an increase in sympathetic activity [4]. The overall incidence of hyperthyroidism in the United States is estimated to be between 0.05% and 1.3%, with a subclinical presentation in a majority of cases [5].

The excess thyroid hormone in the body can have different etiologies. Endogenous causes include Grave’s disease, toxic multinodular goiter, toxic adenoma, TSH-producing adenoma or pituitary adenoma, human chorionic gonadotropin-mediated hyperthyroidism, thyroiditis, and drug-induced disease. While exogenous causes include factitious hyperthyroidism and excessive replacement therapy with levothyroxine [6]. In addition, some medications can have different effects on the thyroid gland itself or act centrally by affecting the hypothalamic-pituitary axis (HPA) and TSH, or peripherally by affecting the T4/T3 transformation. Some of these medications include amiodarone, beta-blockers, interferon-alpha inhibitors, programmed death receptor-1 (PD-1) such as nivolumab and pembrolizumab, and lithium. Epidural steroid injections can also have an impact on the thyroid gland and its hormones by affecting the HPA, though this entity is extremely rare [7]. The theory behind the latter is that epidural steroids directly affect the hypothalamus and the pituitary gland causing an alteration in the hypothalamic-pituitary-thyroid axis, causing an increase in the TSH levels which lead to, eventually, increase in the T4/T3 hormones. This effect can lead to thyrotoxicosis or can worsen an existing condition [7].

Thyrotoxicosis can present with a wide variety of symptoms such as heat intolerance, palpitations, anxiety, fatigue, weight loss, muscle weakness, and irregular menses in women. Clinical findings may include tremor, tachycardia, lid lag, and warm moist skin. The signs and symptoms of subclinical hyperthyroidism, if present, are usually vague and nonspecific [8]. One of the very rare presentations of thyrotoxicosis and hyperthyroidism are hiccups. Hiccups have only been reported in a few case reports as the initial presentation of this disease [9].

Hiccups are common benign involuntary spasmodic and coordinated contractions of the inspiratory muscles associated with a delayed and sudden closure of the glottis which is responsible for the characteristic noise. They are usually transient and resolve spontaneously without treatment [10]. Chronic persistent hiccups are defined as hiccups lasting more than 48 hours and are fairly uncommon. Many disorders can cause hiccups including several neurologic and extraneurologic diseases such as intracranial tumors, strokes, myocardial infarction, renal failure, prostate cancer, or abdominal surgery [2]. Thyrotoxicosis is an extremely rare cause of hiccups. As mentioned earlier, the thyroid hormone affects most of the organs in the body including the hiccups center, which can be irritated by high levels of thyroxine resulting in persistent symptoms [9]. Here, we report a very rare presentation of thyrotoxicosis in a previously undiagnosed gentleman who presented with progressively worsening hiccups. His symptoms started after receiving an epidural steroid injection, which makes our case even more unique. To our knowledge, there is only one reported case in the literature linking steroid injection and the development of thyrotoxicosis with thyrotoxic periodic paralysis and profound hypokalemia. The mechanism of development of hypokalemia after steroid use has been well established but triggering thyrotoxicosis is unknown [7].

Treatment of hyperthyroidism and thyrotoxicosis includes adrenergic symptom relief with beta-blockers. Propranolol also has the theoretical advantage of inhibiting 5′-monodeiodinase, thus blocking the peripheral conversion of T4 to T3 [11]. In addition, hyperthyroidism requires antithyroid pharmacotherapy, radioactive iodine-131 (131I) therapy (the preferred treatment of hyperthyroidism among US thyroid specialists), or thyroidectomy. The treatment modality of choice for hyperthyroidism caused by the overproduction of thyroid hormones depends on the patient’s age, symptoms, comorbidities, and preference [12]. In this report, we have described the case of a gentleman presenting with progressive hiccups as an extremely rare symptom of thyrotoxicosis, triggered by an epidural steroid injection.

## Conclusions

Despite being a rare gastrointestinal manifestation, hiccups can still be the first presenting sign of thyrotoxicosis. Chronic persistent hiccups can be seen in these patients and are likely precipitated by irritation of the hiccup center by high levels of thyroxine. As the treatment of persistent hiccups depends on the underlying cause, it is important to perform a thorough differential diagnosis in these patients. Identifying the etiology and management of hiccups can be challenging in clinical practice and we encourage physicians to expand their differential diagnosis to include a variety of endocrine disorders when treating these patients. Here, we have reported a case of persistent hiccups that cannot be explained by any entity other than thyrotoxicosis.
